# Exploring the ideal combination of activity satisfaction and burden among health promotion volunteers: a cross-sectional study in Japan

**DOI:** 10.1186/1471-2458-13-205

**Published:** 2013-03-07

**Authors:** Hiroshi Murayama, Atsuko Taguchi, Sachiyo Murashima

**Affiliations:** 1 University of Michigan School of Public Health, 1415 Washington Heights, Ann Arbor, MI, 48109-2029, USA; 2 Research Team for Social Participation and Community Health, Tokyo Metropolitan Institute of Gerontology, 35-2 Sakae-cho, Itabashi-ku, Tokyo, 173-0015, Japan; 3 Tohoku University Graduate School of Medicine, 2-1 Seiryo-machi, Aoba-ku, Sendai, Miyagi, 980-8575, Japan; 4 Oita University of Nursing and Health Sciences, 2944-9 Megusuno, Oita City, Oita, 870-1201, Japan

**Keywords:** Health promotion volunteer, Satisfaction, Burden, Activity environment, Japan

## Abstract

**Background:**

Health promotion volunteers (HPVs) who are expected to function as leaders in promoting community health in Japan feel both satisfaction and burden associated with their community engagement activities. The purposes of this study were 1) to describe the prevalence of volunteers with differing levels of activity satisfaction and burden; 2) to examine the association between satisfaction and burden with activity involvement and persistence, and life satisfaction; and 3) to explore associated factors by satisfaction/burden levels among Japanese HPVs. The research question for this study was as follows: What is the relationship between activity satisfaction and burden among HPV?

**Methods:**

A mail-in self-administered questionnaire survey was distributed to 604 HPVs in the cities of Konan and Koka, Shiga Prefecture, central Japan, in September 2005. Questions encompassed demographic data, variables regarding HPV activity such as organizational environment, social support, and the relationship with the neighborhood association, and overall satisfaction and burden related to the activity.

**Results:**

The analyzed sample comprised 422 HPVs. Those with high satisfaction/low burden represented the largest number of study participants (group A; 38.4%). HPVs with high satisfaction/high burden (group B), low satisfaction/low burden (group C), and low satisfaction/high burden (group D) represented 23.0%, 11.1%, and 27.5% of participants, respectively. HPVs in groups A and B reported a greater total number of activities undertaken than those in group C. However, HPVs in group A had higher life satisfaction than those in groups C and D. Multinomial logistic regression analysis used to explore group differences showed that HPVs in group B had lower initial motivation and received less social support from colleagues, and those in group C felt the head of the neighborhood association was uncooperative. Those in group D had lower initial motivation, rated their organizational climate as worse, and considered the head of the neighborhood association uncooperative compared with group A.

**Conclusions:**

We found that feeling satisfied and lightly burdened facilitated HPVs’ active participation in community-based activities. Findings suggest the importance of improving activity environments surrounding HPVs.

## Background

Japan’s rapidly aging population is placing an increasingly serious burden on national agencies because of increased health care costs. Therefore, the importance of health promotion and disease/care prevention strategies in the community has recently become more widely recognized in Japan. One effective way to improve community health and empower the community is to use “natural helpers,” such as community health workers (CHWs) [1,2]. They can offer help in a neighborly way and provide informal and spontaneous assistance [3,4]. Provision of such help could contribute to solving the problems of an aging population. Many earlier studies about CHW activities have revealed behavioral modification; improved health status and health knowledge; access to health support among community members; and more cost-effective health and welfare services as outcomes of CHW interventions. In the past year, review articles on these topics have been published [5-7].

In Japan, health promotion volunteers (HPVs) perform activities similar to those of CHWs [8-10]. The purpose of HPV activities is to maintain and promote the health of community members. HPVs have acted as a natural helping resource in the community for many years. HPV activity originated at the end of World War II. Subsequently, HPV organizations have been established in many municipalities throughout the country to address public health problems most relevant to the times, such as high infant mortality and poor nutrition after the war, and lifestyle-related diseases recently. Like CHWs, they are expected to function as leaders in promoting the health of local residents. HPVs are members of the community who are trained and supported by public health nurses and dietitians affiliated with local municipalities. They are not paid for their services. HPV activities are performed in all areas of the community and take place through organized programs as well as through individual initiatives.

A number of previous studies have reported that volunteer activities provide workers with a sense of fulfillment and personal accomplishment [11,12] as well as increased self-esteem and self-efficacy [13]. In contrast, it has also been reported that some volunteers experience and suffer from emotional overload and burnout because of their activities [11,14]. Thus, working on community activities can affect volunteers both positively and negatively. Moreover, satisfaction and burden related to activities might not be necessarily opposing concepts. There are, in fact, some volunteers who feel both satisfied and burdened when performing their activities. It would be natural to think that those who feel less burdened also experience a higher degree of satisfaction. However, the relationship between satisfaction HPVs derive from their activities and the level of burden they feel has not been clearly delineated in previous studies.

It is important to examine the prevalence of differing levels of activity satisfaction/burden among volunteers and reveal characteristics of HPVs related to satisfaction and burden so that strategies can be developed to support their work. In addition, understanding how levels of satisfaction and burden affect HPVs’ involvement and work persistence in the community is essential. Indeed, the success of workers’ interventions is contingent on their involvement and persistence. It is also important to consider the impact of HPVs’ feelings on their daily lives. Several articles have reported that volunteer work can influence aspects of participants’ quality of life, such as life satisfaction [15-17].

Omoto and Snyder proposed a conceptual framework known as the Volunteer Process Model that specifies psychological and behavioral features associated with each of three sequential stages: antecedents, experiences, and consequences [18]. This model has been modified through empirical research [19-22]. Antecedents include individual variables such as personality, motivation for volunteering, and degree of social support. Experiences include feelings related to volunteer activity such as satisfaction. Finally, consequences include persistence and involvement in the volunteer activity. This model is useful in understanding multiple individual factors and circumstances surrounding volunteers, and in explaining factors that affect feelings about the activity (experiences) and involvement and persistence in the work (consequences), based on a three-stage process.

The purposes of this study were 1) to identify the prevalence of Japanese HPVs according to differing levels of activity satisfaction/burden; 2) to examine the association between satisfaction and burden, volunteers’ activity involvement and persistence, and life satisfaction; and 3) to explore associated factors according to levels of satisfaction/burden among these volunteers. Our specific research question was the following: What is the relationship between activity satisfaction and burden among HPV?

## Methods

### Setting and research participants

We used a cross-sectional design in this study. An anonymous, self-administered questionnaire was mailed directly to all active HPVs in Koka and Konan in September 2005. Participants were all registered in 2005 in the HPV association of either Koka or Konan, two cities in Shiga Prefecture in Japan. Nine HPVs were inactive (six in Koka, three in Konan). There were 604 active HPVs (512 in Koka, 92 in Konan). The HPV organizations of Koka and Konan have often worked in cooperation with each other. In Koka and Konan, the ratio of the population to the number of HPVs was 183.1 and 600.0 (population/HPV), respectively, and the ratio of the area of the city to the number of HPVs was 0.94 and 0.77 (km^2^/ HPV), respectively.

Koka and Konan are adjoining cities in the same county in central Japan, with the community residents of the two cities having culturally similar characteristics. As of September 2005, Koka and Konan had populations of 93,734 and 55,204, respectively. These cities are located within 100 kilometers of Osaka and Nagoya, major cities easily accessible via expressways. Koka and Konan boast farming and traditional ceramics industries. As with many cities, their industrialization began in the 1960s, and, as a result, secondary and tertiary industries began to expand from this period. In 2005, 5.2% of workers in the Koka area were engaged in primary industries, 41.7% in secondary industries, and 53.1% in tertiary industries. In the Konan area, 1.5% of workers were engaged in primary industries, 46.1% in secondary industries, and 52.4% in tertiary industries.

Some people become HPVs on their own initiative, while others are invited to perform this role. To become an HPV, an individual must be nominated by the head of a neighborhood association and attend a municipality-sponsored Health Promotion Volunteer Training Course provided over approximately one year. In this training course, classes provide information concerning health and characteristics of health problems in the locality are described by healthcare professionals (e.g., public health nurses, physicians, and dietitians). Veteran HPVs discuss their experiences in carrying out HPV activities. After completing the course, HPVs are formally commissioned by the mayor. There is no set term of service, and each volunteer usually records her daily activities. The activities of HPVs are funded by the local municipality. Funds are used for the cost of running the activities, and, as mentioned, HPVs are not paid for their labor. They receive advice and support from public health nurses or dietitians who advise them about customary HPV activities and attend their regular meetings.

### Measures

According to the Volunteer Process Model [18-22], we organized the variables into three sequential stages.

#### Antecedent variables

We asked about the process and reasons for the decision to become an HPV, about motivation for engaging in volunteer activities at the end of the HPV Training Course, and about where the volunteers conduct their activities (environment). The decision to become an HPV was considered either passive or active. Motivations for engaging in volunteer activities at the end of the training course were assessed by one item: “I felt highly motivated to engage in HPV activity at the end of the HPV Training Course.” Respondents answered on a four-point Likert scale (1 = strongly disagree, 2 = disagree, 3 = agree, 4 = strongly agree). In the analysis, motivation was classified into two categories: high (3 and 4) and low (1 and 2).

The activity environment included climate of the volunteers’ HPV organization, social support from HPV colleagues and community members, and the relationship between HPVs and the head of the neighborhood association. Climate of the HPV organization was assessed by six items: “HPVs are always friendly to each other in my organization,” “HPVs have a sense of connectedness in my organization,” “HPVs can easily express their opinion in my organization,” “HPVs can ask anything they do not know about the activities without hesitation,” “HPVs are willing to help each other in my organization,” and “HPVs can easily take a break from their activities when they have a personal matter or a job.” Respondents answered on a four-point Likert scale (1 = strongly disagree, 4 = strongly agree). Scores were added, with a higher score indicating a better organizational climate. Cronbach’s alpha was 0.90. In the analysis, we classified data into two categories (good and bad) by median scores.

We assessed two potential sources of social support for HPVs: HPV colleagues (10 items) and community members (6 items). In accordance with the functional classification presented by House [23], items about HPV colleagues included emotional support (e.g., share my satisfaction with and worries about HPV activities), instrumental support (e.g., help me when I find HPV activities burdensome), informational support (e.g., give me information and knowledge about HPV activities), and appraisal support (e.g., provide appreciation and encouragement for my HPV activities). Items about community members were limited to emotional, informational, and appraisal support. Respondents answered on a four-point Likert scale (1 = strongly disagree, 4 = strongly agree). Scores were added up separately for each source of support. The higher the score, the greater was the recognition of availability of social support from each source. Cronbach’s alpha was 0.95 for colleagues and 0.93 for community members. In the analysis, we classified into two categories (high and low) by median scores.

The relationship between HPVs and the head of the neighborhood association was assessed using one item: “In my district, the head of the neighborhood association is uncooperative regarding HPV activities.” Respondents answered on a four-point Likert scale (1 = strongly disagree, 4 = strongly agree). In the analysis, we classified data into two categories: agree (3 and 4) and disagree (1 and 2).

#### Experience variables

We included overall activity satisfaction and burden, as assessed by two items. Participants responded to the statement “Overall, I am satisfied with my HPV activity,” using a four-point Likert scale (1 = strongly disagree, 4 = strongly agree). The other item was “Overall, I feel burdened by my HPV activity” and was scored using the same four-point Likert scale. Satisfaction and burden were dichotomized: high (3 and 4) and low (1 and 2).

#### Consequence variables

Activity involvement and persistence were included, with proxies of these being the number of activities an HPV performed over a 3-month period and years of HPV experience, respectively. The 3-month period we selected was considered usual in that no special event related to HPV activities occurred during that time. In this study, in addition to consequence factors regarding HPV activity, we also included quality of life as a consequence of HPVs’ daily lives. This was assessed by one item for life satisfaction: “I am satisfied with my daily life,” rated on a four-point Likert scale (1 = strongly disagree, 4 = strongly agree). In the analysis, responses were classified into two categories: high (3 and 4) and low (1 and 2).

#### Demographics

We inquired about gender, age, socioeconomic status, neighborly ties, interest in being a volunteer, and self-rated health. Socioeconomic status included employment and educational level. Employment was classified according to two categories: employed (full-time, self-employed/agriculture, or part-time) and unemployed. Educational level was also classified into two categories: higher education (completed junior college, vocational school, college, or graduate school) and lower education (completed junior high or high school). Neighborly ties were determined by one item, and respondents answered on a four-point Likert scale (1 = almost no ties, 2 = weak ties, 3 = somewhat strong ties, 4 = strong ties). Responses were classified into two categories: strong (3 and 4) and weak (1 and 2).

### Statistical analysis

First, according to their answers regarding activity satisfaction and burden, we categorized the participants into four groups: high satisfaction and low burden (hereafter group A), high satisfaction and high burden (hereafter group B), low satisfaction and low burden (hereafter group C), and low satisfaction and high burden (hereafter group D). Moreover, we compared demographics and antecedent variables among the four groups, organized by satisfaction and burden, using a one-way analysis of variance for the continuous scales, the Kruskal-Wallis test for the ordinal scale, and the chi-square test for the nominal scale.

Second, we compared consequence variables among the four groups. A generalized linear model was used to examine the differences, setting consequence variables as the response variables. The total number of activities and years of experience were used as continuous scales, and life satisfaction was a nominal scale. We used age, initial motivation, and affiliation (city) as covariates. Although we analyzed the combined data for Koka and Konan, we adjusted for the difference in city area between the two using the variable of affiliation in the model because the ratio of population to number of HPVs was different between the two cities.

Finally, to investigate which factors were associated with differences among HPV groups when divided by levels of satisfaction and burden (experience variables), a multinomial logistic regression analysis was performed. Demographics and antecedent variables that were significantly different among the four groups were selected as independent variables and included in the model. In addition, affiliation was adjusted for in the model. Results were calculated as adjusted odds ratios (ORs) with 95% confidence intervals (95% CIs). A two-tailed p < 0.05 was considered significant, with p < 0.10 considered marginally significant. We used IBM SPSS 20.

## Results

A total of 604 questionnaires were distributed, and 433 were returned (response rate, 71.7%). After excluding 11 questionnaires that did not have answers regarding activity satisfaction and/or burden, 422 were used for the analysis (valid response rate, 69.9%). Of these questionnaires, 352 were from Koka, and 70 were from Konan (valid response rates, 68.8% and 76.1%, respectively). All HPVs who participated in this study were women, and mean age was 54.2 years. Approximately 70% graduated from junior high school or high school (categorized as lower education in this study).

Regarding the distribution of the four groups by activity satisfaction and burden, group A (HPVs with high satisfaction and low burden) comprised the largest number of study participants (n = 162, 38.4%). Group B represented approximately a quarter of the participants (HPVs with high satisfaction and high burden; n = 97, 23.0%). Group D was a little larger than group B (HPVs with low satisfaction and high burden; n = 116, 27.5%). The smallest was group C (HPVs with low satisfaction and low burden; n = 47, 11.1%). Table 1 shows the characteristics of the four groups. HPVs in group A were the oldest. HPVs in groups A and B had better self-rated health than those in groups C and D. The initial motivation of HPVs in groups A and C was higher than that in groups B and D. Regarding HPV organizational environments, HPVs in group A evaluated their organizations more positively than those in the other groups.

**Table 1:** Characteristics of participants by levels of activity satisfaction and burden

			**Total**	**Group A**	**Group B**	**Group C**	**Group D**	***p***	**Group difference** **(*p *< 0.05)**
				**High satisfaction-low burden**	**High satisfaction-high burden**	**Low satisfaction-low burden**	**Low satisfaction-high burden**		
			**N = 422**	**n = 162**	**n = 97**	**n = 47**	**n = 116**		
**Demographics**									
	Affiliation	Koka city	353 (83.6)	131 (80.9)	77 (79.4)	42 (89.4)	102 (87.9)	0.186	
	Age		54.2 ± 6.8	56.0 ± 6.4	52.4 ± 7.3	54.6 ± 7.0	53.2 ± 6.1	<0.001	A-B, A-D
	Unemployed		144 (34.1)	65 (40.1)	31 (32.0)	17 (36.2)	31 (26.7)	0.187	
	Educational level	Low	292 (69.2)	114 (70.4)	69 (71.1)	32 (68.1)	77 (66.4)	0.983	
	Neighborly ties	Weak	21 (5.0)	6 (3.7)	4 (4.1)	2 (4.3)	9 (7.8)	0.383	
	Interest in being a volunteer	Low	84 (19.9)	23 (14.2)	21 (21.6)	4 (8.5)	36 (31.0)	0.001	
	Self-rated health	Poor	48 (11.4)	14 (8.6)	7 (7.2)	6 (12.8)	21 (18.1)	0.027	
**Antecedents**									
	Reasons for becoming an HPV	Passive	340 (80.6)	130 (80.2)	81 (83.5)	32 (68.1)	97 (83.6)	0.115	
	Motivation at the end of the HPV training course	Low	99 (23.5)	15 (9.3)	30 (30.9)	5 (10.6)	49 (42.2)	<0.001	
	Climate of the HPV organization	Bad (below median)	179 (42.4)	45 (27.8)	40 (41.2)	18 (38.3)	76 (65.5)	<0.001	
	Social support from colleagues	Low (below median)	264 (62.6)	80 (49.4)	69 (71.1)	29 (61.7)	86 (74.1)	<0.001	
	Social support from community members	Low (below median)	242 (57.3)	72 (44.4)	54 (55.7)	31 (66.0)	85 (73.3)	<0.001	
	Head of the neighborhood association uncooperative regarding HPV activities	Agree	134 (31.8)	29 (17.9)	33 (34.0)	18 (38.3)	54 (46.6)	<0.001	

Values represent mean ± SD or n (%).

Table 2 provides a comparison among the groups in total number of activities undertaken, years of experience, and life satisfaction, defined as the consequence variables in this study. HPVs in group B reported the greatest total number of activities undertaken; those in group A reported the second largest number. The generalized linear model showed significant differences between groups A and C and between groups B and C. Regarding life satisfaction, HPVs in group A were the most satisfied with their lives, with significant differences between groups A and C and between groups A and D. In contrast, there was no significant difference in years of experience among the four groups.

**Table 2:** Association of activity satisfaction/burden levels with consequences of HPV activity: generalized linear model

			**Total**	**Group A**	**Group B**	**Group C**	**Group D**	***p***	**Group difference** **(*p *< 0.05)**
				**High satisfaction-low burden**	**High satisfaction-high burden**	**Low satisfaction-low burden**	**Low satisfaction-high burden**		
**Activity involvement**									
	Total number of activities during a 3-month period		7.3 ± 7.2 (Median: 5)	7.6 ± 7.5 (Median: 6)	8.5 ± 8.3 (Median: 6)	5.2 ± 3.5 (Median: 4.5)	6.4 ± 6.6 (Median: 5)	0.026	A-C, B-C
**Activity persistence**									
	Years of experience		6.4 ± 5.2 (Median: 5)	6.8 ± 5.9 (Median: 5)	6.5 ± 4.5 (Median: 5)	6.3 ± 6.3 (Median: 4)	5.7 ± 4.4 (Median: 4)	0.429	
**Quality of life**									
	Life satisfaction	Low	47 (11.1)	6 (3.7)	11 (11.3)	6 (12.8)	24 (20.7)	0.015	A-C, A-D

Values represent mean ± SD or n (%).

Age, initial motivation, and affiliation (city) were used as covariates.

Table 3 shows the results of the multinomial logistic regression analysis examining associated factors among HPV groups by satisfaction/burden levels. We set group A (HPVs with high satisfaction and low burden) as a reference group because this was the largest group of study participants. Variables that were significantly different among the four groups in Table 1 (age, interest in being a volunteer, self-rated health, motivation at the end of the HPV training course, climate of the HPV organization, social support from colleagues, social support from community members, and the relationship with the head of the neighborhood association) and affiliation were adjusted for in the model. The HPVs in group B were younger, had lower initial motivation, and received less social support from colleagues than those in group A. Group C participants generally felt the head of the neighborhood association was uncooperative compared with those in group A. HPVs in group D were younger; had poorer self-rated health and lower initial motivation; and felt their organizational climate was worse, with the head of the neighborhood association being uncooperative, in contrast to group A.

**Table 3:** Factors associated with levels of activity satisfaction/burden in HPVs: multinomial logistic regression analysis

		**Group B**		**Group C**		**Group D**	
		**High satisfaction-high burden**		**Low satisfaction-low burden**		**Low satisfaction-high burden**	
		**OR**	**(95% CI)**	**OR**	**(95% CI)**	**OR**	**(95% CI)**
**Demographics**							
Age	(Every 5 years)	0.67^**^	(0.53–0.85)	0.84	(0.63–1.12)	0.75^*^	(0.59–0.96)
Interest in being a volunteer	Low	1.25	(0.68–2.30)	0.96	(0.45–2.07)	1.59	(0.85–2.96)
Self-rated health	Poor	0.90	(0.31–2.58)	1.68	(0.52–5.41)	2.61^*^	(1.05–6.51)
**Antecedents**							
Motivation at the end of the HPV training course	Low	3.65^**^	(1.74–7.66)	0.76	(0.23–2.50)	4.94^***^	(2.35–10.36)
Climate of the HPV organization	Bad	1.73^†^	(0.92–3.25)	1.45	(0.66–3.19)	3.91^***^	(2.05–7.49)
Social support from colleagues	Low	2.08^*^	(1.09–3.97)	1.18	(0.54–2.59)	1.32	(0.66–2.62)
Social support from community members	Low	0.89	(0.47–1.68)	1.60	(0.71–3.59)	1.96^†^	(0.98–3.93)
Head of the neighborhood association uncooperative regarding HPV activities	Agree	1.88^†^	(0.92–3.84)	2.70^*^	(1.17–6.26)	2.50^*^	(1.24–5.04)

OR: odds ratio. 95% CI: 95% confidence interval.

^***^p < 0.001. ^**^p < 0.01. ^*^p < 0.05. ^†^*p* < 0.10.

Reference category for dependent variable was group A (high satisfaction-low burden).

Age, interest in being a volunteer, self-rated health, motivation at the end of the HPV training course, climate of the HPV organization, social support from colleagues, social support from community members, the relationship with the head of the neighborhood association, and affiliations (city) were adjusted for in the model.

## Discussion

This study aimed to understand trends associated with Japanese HPVs through an analysis of satisfaction level and perceived burden, to examine how levels of satisfaction/burden affect them in their daily lives, and to explore factors associated with different levels. We obtained three main findings in accordance with our research purposes.

First, HPVs who enjoyed high satisfaction coupled with a low level of perceived burden made up the largest group (38.4%). However, it was notable that other combinations of satisfaction/burden levels were also prevalent. Because the response rate for this survey was good, we posit that the results realistically reflect actual HPV conditions. Daniels et al. implied the coexistence of positive feelings such as accomplishment, and negative feelings such as suffering in lay health workers because of the dual demands of caring for their families and working on community activities [11]. Similarly, satisfaction and burden related to HPV activities are not mutually exclusive. Understanding the characteristics of each group is important for organizational management. Surprisingly, approximately a quarter of participants (27.5%) were categorized into group D. Although HPVs in this group had low satisfaction and high burden, their years of experience did not statistically differ from those in the other groups. HPV activities are performed in all areas of the community, indicating the importance of organized programs as well as individual initiatives. However, major difficulties that HPVs experienced in their daily work included recruitment of new members and a decrease in number of members who could flexibly engage in activities because of an increasing number who had jobs [24]. Therefore, there may be times when HPVs cannot easily leave their volunteer work, even though they feel less satisfied and experience burden related to their activities.

Second, HPVs who were satisfied with their activities (groups A and B) had greater activity involvement, and those with high satisfaction and low burden (group A) had greater life satisfaction. In terms of activity involvement, being satisfied with their activities was important, whether or not HPVs felt the activity was a burden. This result is consistent with findings from a study of volunteers using the Volunteer Process Model [19]. However, from the aspect of quality of life, HPVs’ satisfaction with the activity as well as less perceived burden is even more desirable. Several longitudinal studies have reported that greater involvement in volunteer activities often increases the participants’ well-being and life satisfaction [15-17]. Both activity satisfaction and burden were possible confounding factors between activity involvement and life satisfaction. To our knowledge, there are no studies that have examined the association between levels of activity satisfaction and burden with quality of life among volunteer workers. We conclude that having high satisfaction and low burden related to the activity has a beneficial effect on not only activity involvement but also the volunteers’ quality of life, although an examination of causal relationships among these variables should be conducted in future studies.

Third, to understand the characteristics of the groups, we explored associated factors for each, setting group A as a reference group. We found that environmental factors related to HPV activities such as a more facilitative organizational climate, increased social support, and a more cooperative relationship with the neighborhood association were associated with high volunteer satisfaction and low burden (group A), as well as individual factors such as higher initial motivation. This implies that improvement of the activity environment increases HPVs’ satisfaction and decreases their sense of burden associated with the activity. Their relationship with the neighborhood association appears to be particularly important. HPVs in group A enjoyed a more cooperative relationship with the head of the neighborhood association compared with those in other groups. Because HPVs engage in community-based health promotion activities, volunteers are expected to mobilize community members and resources [3,4,25]. For HPVs to meet such expectations regarding their role, the relationship with the head of the neighborhood association is arguably highly important. Therefore, HPVs who enjoy a good relationship with that person feel more satisfaction and less burden related to their activities.

HPVs in groups B and D had lower initial motivation for activities than those in group A. Previous studies on HPV activity reported the importance of increasing initial motivation to enhance activity persistence and satisfaction, thereby decreasing perceived burden and strengthening community health outreach initiatives [8-10]. In addition to improving the organizational environment, the development of strategies to train highly motivated new volunteers is essential in energizing current HPVs.

Overall, we discovered one main, overriding answer to our research question. The ideal situation for HPVs is experiencing a high level of satisfaction coupled with a low level of self-perceived burden when participating in an activity. This is illustrated by the results in Table 2. Although this finding might seem obvious, until now there have been no studies undertaken to support this hypothesis. The study also showed that HPVs in the other three groups, in addition to HPVs in group A (high satisfaction/low burden), were present in each organization. Thus, it is important to assess the prevalence of members with differing individual satisfaction/burden levels in each organization and set a high-priority target group for intervention. Moreover, based on the characteristics of each group, development of strategies for each one could be effective.

Several limitations of our research should be noted. First, this was a cross-sectional study. Hence, we cannot explain causal relationships. In this study, we set the variables based on the Volunteer Process Model. However, a longitudinal study design could provide more robust and useful evidence. Second, we used combined data from Koka and Kona because the HPV organizations of those cities work in cooperation with each other. However, we adjusted for the differences between the two cities in the analysis. Third, this study was conducted only on Japanese HPVs, with the target HPV organizations limited to those in two areas. The activities of HPVs in Japan are similar to those performed by CHWs in the United States, but the cultural backgrounds of communities are different between the two countries. Although this study provides clear evidence regarding ways to encourage HPVs, to increase the generalizability of the findings future studies should include different settings (e.g., different countries or municipalities with different cultural backgrounds). Fourth, the study data were collected in 2005. Although there have not been any significant changes in HPV activities related to policy, organizational management, or their role in the community, we must carefully interpret the findings in this study, taking into consideration the data collection period.

## Conclusions

Grassroots HPV activities have the potential to promote the health of the community. We confirmed that HPVs who feel satisfied and lightly burdened are instrumental in promoting HPV community-based activities. In addition, our study suggests the importance of improving activity environments surrounding HPVs. Thus, it is important to assess levels of activity satisfaction and burden among HPVs and develop policies that contribute to building better activity environments.

## Consent

For this study, an anonymous, self-administered questionnaire was mailed directly to the participants. A statement attached to the questionnaire explained the purpose of the study, that participation was voluntary, and a promise of anonymity. Return of the questionnaire was taken as consent to participate in the survey. The survey was explained to and approved by the HPV associations of Koka and Konan and the municipalities of both cities. The study was conducted after obtaining approval from the Ethics Committee of the Graduate School of Medicine and Faculty of Medicine at The University of Tokyo (No. 1182, in 2005).

## Competing interests

The authors declare that they have no competing interests.

## Authors’ contributions

HM conceived of the study, performed data collection and data analysis, and drafted the manuscript. AT conceived of the study, performed data collection and contributed to the interpretation of the results. SM conceived of the study and contributed to the interpretation of the results. All authors read and approved the final manuscript.

## Pre-publication history

The pre-publication history for this paper can be accessed here:

http://www.biomedcentral.com/1471-2458/13/205/prepub

## References

[B1] Eng E Rhodes SD Parker E Diclemente RJ, Crosby RA, Kegler MC Natural helper models to enhance a community’s health and competence Emerging theories in health promotion practice and research 2002 2 CA: Jossey-Bass 303 330

[B2] Haines A Sanders D Lehmann U Rowe AK Lawn JE Jan S Walker DG Bhutta Z Achieving child survival goals: potential contribution of community health workers Lancet 2007 369 2121 2131 10.1016/S0140-6736(07)60325-0 17586307

[B3] Earp JA Flax VL What lay health advisors do: an evaluation of advisors’ activities Canc Pract 1999 7 16 21 10.1046/j.1523-5394.1999.07104.x 9892999

[B4] Eng E Young R Lay health advisors as community change agents Fam Community Health 1992 15 24 40 10.1097/00003727-199204000-00005

[B5] Ayala GX Vaz L Earp JA Elder JP Cherrington A Outcome effectiveness of the lay health advisor model among Latinos in the United States: an examination by role Health Educ Res 2010 25 815 840 10.1093/her/cyq035 20603384 PMC2948840

[B6] Lewin S Munabi-Babigumira S Glenton C Daniels K Bosch-Capblanch X van Wyk BE Odgaard-Jensen J Johansen M Aja GN Zwarenstein M Scheel IB Lay health workers in primary and community health care for maternal and child health and the management of infectious diseases Cochrane Database Syst Rev 2010 3 10.1002/14651858.CD004015.pub3 PMC6485809 20238326

[B7] Viswanathan M Kraschnewski JL Nishikawa B Morgan LC Honeycutt AA Thieda P Lohr KN Jonas DE Outcomes and costs of community health worker intervention: a systematic review Med Care 2010 48 792 808 10.1097/MLR.0b013e3181e35b51 20706166

[B8] Murayama H Taguchi A Murashima S Differences in psychosocial factors among novice, experienced, and veteran health promotion volunteers in Japan Publ Health Nurs 2008 25 253 260 10.1111/j.1525-1446.2008.00702.x 18477376

[B9] Murayama H Taguchi A Murashima S The relationships between feelings of satisfaction and burden with respect to activity and social support among health promotion volunteers in Japan Health Educ Behav 2010 37 275 287 10.1177/1090198109341782 19843687

[B10] Murayama H Taguchi A Murashima S Exploring strategies to encourage community health outreach by health promotion volunteers in Japan J Ambul Care Manage 2011 34 274 285 21673525 10.1097/JAC.0b013e318223f427

[B11] Daniels K Van Zyl HH Clarke M Dick J Johansson E Ear to the ground: listening to farm dwellers talk about the experience of becoming lay health workers Health Pol 2005 73 92 103 10.1016/j.healthpol.2004.10.006 15911060

[B12] Ramirez-Valles J Changing women: the narrative construction of personal change through community health work among women in Mexico Health Educ Behav 1999 26 25 42 10.1177/109019819902600104 9952050

[B13] Rodriguez VM Conway TL Woodruff SI Edwards CC Pilot test of an assessment instrument for Latina community health advisors conducting an ETS intervention J Immigr Health 2003 5 129 137 10.1023/A:1023991818829 14512767

[B14] Yan EC Tang CS The role of individual, interpersonal, and organizational factors in mitigating burnout among elderly Chinese volunteers Int J Geriatr Psychiatr 2003 18 795 802 10.1002/gps.922 12949847

[B15] Menec VH The relation between everyday activities and successful aging: a 6-year longitudinal study J Gerontol Ser B 2003 58 S74 S82 10.1093/geronb/58.2.S74 12646596

[B16] Thoits PA Hewitt LN Volunteer work and well-being J Health Soc Behav 2001 42 115 131 10.2307/3090173 11467248

[B17] Van Willigen M Differential benefits of volunteering across the life course J Gerontol Ser B 2000 55 S308 S318 10.1093/geronb/55.5.S308 10985302

[B18] Omoto AM Snyder M Sustained helping without obligation: Motivation, longevity of service, and perceived attitude change among AIDS volunteers J Pers Soc Psychol 1995 68 671 686 7738770 10.1037//0022-3514.68.4.671

[B19] Davis MH Hall JA Meyer M The first year: influences on the satisfaction, involvement, and persistence of new community volunteers Pers Soc Psychol Bull 2003 29 248 260 10.1177/0146167202239050 15272952

[B20] Omoto AM Snyder M Hackett JD Personality and motivational antecedents of activism and civic engagement J Pers 2010 78 1703 1734 10.1111/j.1467-6494.2010.00667.x 21039529

[B21] Omoto AM Snyder M Martino SC Volunteerism and the life course: investigating age-related agendas for action Basic Appl Soc Psychol 2000 22 181 198

[B22] Snyder M Omoto AM Volunteerism: social issues perspectives and social policy implications Soc Issues Pol Rev 2008 2 1 36 10.1111/j.1751-2409.2008.00009.x

[B23] House JS Work Stress and Social Support 1981 Reading, MA: Addison-Wesley

[B24] Murayama H Taguchi A Murashima S Ryu S Levels of the consciousness of activities among health promotion volunteers: comparison by years of volunteer’s experience Jpn J Publ Health 2007 54 633 643 Japanese with English abstract 17972434

[B25] Johnson RE Green BL Anderson-Lewis C Wynn TA Community health advisors as research partners: an evaluation of the training and activities Fam Community Health 2005 28 41 50 10.1097/00003727-200501000-00007 15625505

